# Multi-AUV Dynamic Cooperative Path Planning with Hybrid Particle Swarm and Dynamic Window Algorithm in Three-Dimensional Terrain and Ocean Current Environment

**DOI:** 10.3390/biomimetics10080536

**Published:** 2025-08-15

**Authors:** Bing Sun, Ziang Lv

**Affiliations:** Logistics Engineering College, Shanghai Maritime University, Shanghai 201306, China; 202330210091@stu.shmtu.edu.cn

**Keywords:** multi-AUVs, ocean current, particle swarm optimization, dynamic path planning, dynamic window algorithm

## Abstract

Aiming at the cooperative path-planning problem of multiple autonomous underwater vehicles in underwater three-dimensional terrain and dynamic ocean current environments, a hybrid algorithm based on the Improved Multi-Objective Particle Swarm Optimization (IMOPSO) and Dynamic Window (DWA) is proposed. The traditional particle swarm optimization algorithm is prone to falling into local optimization in high-dimensional and complex marine environments. It is difficult to meet multiple constraint conditions, the particle distribution is uneven, and the adaptability to dynamic environments is poor. In response to these problems, a hybrid initialization method based on Chebyshev chaotic mapping, pre-iterative elimination, and boundary particle injection (CPB) is proposed, and the particle swarm optimization algorithm is improved by combining dynamic parameter adjustment and a hybrid perturbation mechanism. On this basis, the Dynamic Window Method (DWA) is introduced as the local path optimization module to achieve real-time avoidance of dynamic obstacles and rolling path correction, thereby constructing a globally and locally coupled hybrid path-planning framework. Finally, cubic spline interpolation is used to smooth the planned path. Considering factors such as path length, smoothness, deflection Angle, and ocean current kinetic energy loss, the dynamic penalty function is adopted to optimize the multi-AUV cooperative collision avoidance and terrain constraints. The simulation results show that the proposed algorithm can effectively plan the dynamic safe path planning of multiple AUVs. By comparing it with other algorithms, the efficiency and security of the proposed algorithm are verified, meeting the navigation requirements in the current environment. Experiments show that the IMOPSO–DWA hybrid algorithm reduces the path length by 15.5%, the threat penalty by 8.3%, and the total fitness by 3.2% compared with the traditional PSO algorithm.

## 1. Introduction

Autonomous Underwater Vehicles (AUVs) have become indispensable tools in modern marine applications such as oceanographic surveys, environmental monitoring, seabed mapping, and search-and-rescue operations [1]. The emergence of multi-AUV systems has significantly enhanced mission efficiency and fault tolerance by enabling distributed task execution. However, planning safe, efficient, and cooperative paths for multiple AUVs in a dynamic underwater environment remains a major challenge. This problem is inherently high-dimensional, nonlinear, and constrained, often involving multiple objectives such as minimizing energy consumption, avoiding inter-vehicle collisions, evading obstacles, and maximizing task coverage [2].

The proposed path-planning methods at present can be roughly divided into four major categories. Classic graph-based algorithms, such as A* [3] and Dijkstra [4], are still widely used because they can calculate the global optimal path on discrete mappings. However, their computing costs limit their applicability in large-scale or real-time multi-AUV systems. Sampling-based methods, including Probabilistic Roadmap [5] and Rapidly Exploring Random Trees [6], can effectively explore high-dimensional space. However, they often generate suboptimal or unstable paths and have limited adaptability to dynamic environments. Based on optimization methods, such as nonlinear programming [7] and dynamic programming [8], continuous control with constraints and precise modeling is provided, but they have the disadvantages of high dimensionality and high computational cost. This makes them unsuitable for online or multi-agent scenarios. In addition, optimization-based algorithms also include meta-heuristic algorithms inspired by nature, such as genetic algorithms [9], ant colony optimization [10], and particle swarm optimization [11]. Due to their flexibility and strong global search capabilities, they are widely popular. In the past two years, a variety of new meta-heuristic methods have emerged. For example, the IMOSBOA proposed by Tang et al. [12] addresses environmental and threat uncertainties in AUV 3D path planning, while the AI-SMA proposed by Ma et al. [13] effectively improves the performance of UAV swarm path planning through an adaptive perturbation strategy. Among them, the particle swarm optimization algorithm has the advantages of simplicity and fast convergence, and is particularly suitable for multi-AUV path planning.

In recent years, machine learning methods have shown increasing potential in path planning in complex environments. Puente-Castro et al. [14] proposed an efficient path-planning method for unmanned aerial vehicle (UAV) swarms with dense obstacles based on Q-learning. Liu et al. [15] designed a deep reinforcement learning framework that combines hybrid heuristic search with policy learning, which improves the quality of real-time paths and control efficiency. Tao et al. [16] introduced an adaptive soft Actor-Critic architecture for local path planning in dynamic environments, emphasizing policy adaptability and obstacle-aware decision-making to ensure smooth and collision-free trajectories.

Recent studies have improved the particle swarm optimization algorithm. Babakhani et al. [17] combined the penalty function with particle swarm optimization technology, which can effectively handle the complex constraints in real marine environments and improve the applicability and accuracy of AUV motion planning. However, its penalty function parameters are highly sensitive, and its application effect in high-dynamic marine environment scenarios may be limited. Wang et al. [18] integrated PSO with the adaptive step-size cuckoo search algorithm, taking into account both global optimization ability and local search accuracy, thereby enhancing the efficiency and robustness of path planning for AUVs in the complex 3D underwater environment. However, its adaptive step-size parameter adjustment mechanism is rather complex, and the convergence speed in high-density obstacle scenarios needs to be optimized. Zhi et al. [19] proposed AMP-PSO, which divided particles into dominant subgroups and follower subgroups and introduced an inter-group exchange mechanism to handle multi-AUV cooperative tasks. It outperformed the classical PSO and other improved methods in simulation, but the mechanism was complex, with many parameters and great difficulty in parameter adjustment. Wang et al. [20] combined Levy flight and orthogonal learning strategies with PSO to systematically mine population historical information. Their optimization accuracy was improved by 2–3 orders of magnitude compared to traditional PSO, and the convergence speed was accelerated by 40%. However, the robustness in dynamic environments still needs to be verified. The random jump characteristic of Levy’s flight may lead to a decrease in path smoothness.

Despite these advantages, standard PSOs lack real-time response capabilities, which are crucial in dynamic underwater environments. For dynamic environments with real-time constraints, online optimization techniques have certain advantages in terms of responsiveness. The Model Predictive Control (MPC) framework achieves rolling horizontal re-programming by solving constrained optimization problems within a limited time window. Liu et al. [21] proposed a real-time reward collection framework for unmanned aerial vehicles based on online optimization, achieving dynamic obstacle response optimization through moving time-domain approximation. Chen et al. [22] proposed a trajectory planning scheme based on online optimization for the problem of heterogeneous multi-robot cooperative landing tasks. Combined with the MPC framework, the optimization problem was solved by rolling in the finite time domain, achieving the real-time replanning capability with high responsiveness. In addition, the dynamic window method [23] has the characteristics of low computational cost and strong real-time performance. It calculates feasible motion instructions within a limited time window by evaluating dynamic constraints and obstacle distances. The adaptability of DWA to underwater robots includes water flow sensing planning [24], real-time obstacle avoidance [25], and cooperative tracking in dynamic environments.

In parallel, the rise of biomimetics—the design and application of systems modeled on biological entities—has injected new vitality into the development of intelligent underwater robotics. Marine animals exhibit remarkable efficiency, adaptability, and robustness in navigating complex aquatic environments. The schooling behavior of fish, the hunting strategies of cephalopods, and the long-distance foraging patterns of seabirds have inspired new methods for group coordination, collision avoidance, and energy-efficient movement in multi-AUV systems. By mimicking these natural behaviors, engineers have developed control strategies and path-planning algorithms that capitalize on the emergent intelligence and adaptability inherent in biological systems.

Among bioinspired techniques, Levy flight [26]—a random walk pattern observed in the foraging behavior of animals like albatrosses and fruit flies—has gained traction for its capacity to balance global exploration with local exploitation in optimization tasks. When integrated with swarm intelligence frameworks such as PSO, Levy flight enhances the algorithm’s ability to escape local optima and explore diverse solution spaces [27]. Additionally, biologically inspired mechanisms like differential mutation and adaptive learning from collective behaviors have further improved swarm diversity and convergence performance in dynamic, high-dimensional environments. These biomimetic enhancements not only provide theoretical grounding for improved performance but also offer practical advantages for path planning in the unpredictable and obstacle-rich domains where AUVs operate.

This paper proposes a hybrid PSO-DWA framework that integrates global trajectory optimization with real-time local trajectory planning. The uniqueness of the PSO improvement strategy proposed in this paper is reflected in three aspects: Firstly, compared with Zhang et al. [28] who only rely on real-time adjustment of ocean currents, this paper adopts a hybrid initialization mechanism that integrates Chebyshev chaotic mapping, pre-iterative transient elimination, and boundary particle injection to solve the problem of uneven particle distribution in high-dimensional terrain in traditional PSO. Secondly, a dynamic parameter adaptive mechanism is introduced to overcome the response lag of static parameters, such as Lin et al. [29] in dynamic obstacle scenarios by introducing an inertia weight index attenuation and asymmetric learning factors. Finally, to avoid the convergent oscillation problem caused by the single Levy strategy adopted by Sutantyo et al. [30], this paper innovatively employs a hybrid perturbation strategy combining Levy flight and differential variation. A multi-objective DWA cost function is designed by integrating obstacle distance, trajectory smoothness, and energy efficiency metrics. Additionally, collaborative strategies such as track prediction, communication topology management, and coordinated collision avoidance are employed to ensure safe and efficient multi-AUV path planning.

## 2. Underwater Environment Modeling

### 2.1. Underwater Terrain Environment Modeling

Compared with the land topography formed by crustal movement and weathering erosion, the seabed topography is mainly influenced by seabed volcanic activities and deep-sea sedimentation. Although there are many differences between the seabed topography and the land topography in terms of geomorphic features and environmental conditions, the two still have certain similarities in aspects such as formation mechanisms. Therefore, the land-based mountain model can be used to represent the seabed topography [31].

The complex terrain environment of this study consists of the reference terrain and various types of static obstacles. The modeling of the reference terrain is generated by superimposing multi-scale waveforms, namely trigonometric functions. Meanwhile, to simulate the irregularity of the real terrain, Gaussian white noise is superimposed, and the noise intensity is amplified to simulate the terrain features covering different scales [32]. Its mathematical description is as follows:

$$\begin{array}{cc} H_{base}\left(x,y\right)= & sin\left(y+10\right)+0.2sin\left(x\right)+0.1cos\left(0.6x^{2}+y^{2}\right)\\ \; & +1.1cos\left(y\right)+sin\left(0.1x^{2}+y^{2}\right)+N\left(0,\sigma^{2}\right) \end{array}$$

where (sin(y+10)) represents the longitudinal main wave, the periodic undulation of the dominant terrain along the Y-axis direction, and a phase offset of 10 units simulates asymmetric terrain. (0.2sin(x)) represents the lateral fine-tuning wave, superimposing small amplitude fluctuations in the X-axis direction to form lateral micro-terrain changes. 0.1cos(0.6r) represents radial mesoscale fluctuations, annular ripples centered on the origin, and simulates moderate-scale terrain undulations. (1.1cos(y)) represents the longitudinal cosine wave. To enhance the symmetry of the terrain in the Y-axis direction and form an alternating ridge and valley structure. (sin(0.1r)) represents global low-frequency ripples, long-wavelength global fluctuations, and simulates macroscopic terrain trends. $N(0,\sigma^{2})$ represents the Gaussian distribution random field. The noise amplitude is approximately 200% of the main wave amplitude of the terrain, significantly increasing the microscopic roughness.

Reference [33] proposes an enhanced dynamic artificial potential field method for the three-dimensional path planning of multi-rotor unmanned aerial vehicles. Terrain modeling is carried out using the sine wave superposition model. However, the sine wave superposition model is more suitable for simulating a large-scale and periodically changing environment. Therefore, the following mathematical model is adopted in this paper to describe the submarine mountains:

(1) $$H_{peak}^{i}\left(x,y\right)=h_{i}^{p}\cdot exp\left(-\frac{{\left(x-x_{i}^{p}\right)}^{2}}{2{\left(\sigma_{i}^{x}\right)}^{2}}-\frac{{\left(y-y_{i}^{p}\right)}^{2}}{2{\left(\sigma_{i}^{y}\right)}^{2}}\right)$$

where $H_{peak}^{i}\left(x,y\right)$ represents the terrain height distribution function of the obstacle on the i-th mountain peak; $\left(x_{i}^{p},y_{i}^{p}\right)$ represents the center coordinates of the mountain peak, $h_{i}^{p}$ is the peak height of the ith mountain peak in meters, $\sigma_{i}^{x}$ and $\sigma_{i}^{y}$ are the attenuation coefficients in the x-direction and y-direction, respectively, which are used to control the steepness of each layer of the mountain ground.

Furthermore, set up a height model of the composite terrain. A highly complex and high-fidelity virtual seabed terrain environment is constructed by nonlinearly superimposing the basic terrain features of multiple Gaussian mixture models [34]. The formula is as follows:

(2) $$H_{total}\left(x,y\right)=max\left(H_{base}\left(x,y\right),\max_{i=1}^{N_{m}}H_{peak}^{i}\left(x,y\right)\right)+N\left(0,0.5\right))$$

where $H_{base}$ is the reference terrain elevation, and the mountain peak area forms natural obstacles through elevation superposition. $H_{peak}^{i}$ is the height field of the i-th obstacle, and N is the Gaussian noise introduced for superposition to simulate the roughness of the seabed.

### 2.2. Obstacle Modeling

In addition to specific submarine topography modeling, obstacles, as impassable or evasive areas in the environment, directly affect the feasibility and superiority of the path. Obstacles can be roughly divided into static obstacles and dynamic obstacles.

#### 2.2.1. Static Obstacle Modeling

**Figure 1:**
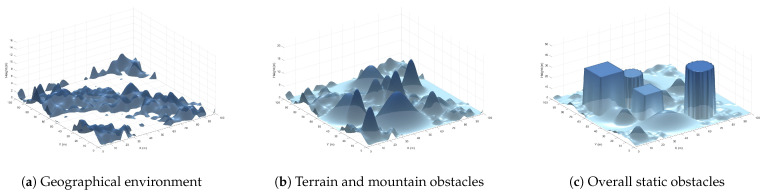
Overview of the environmental model.

Static obstacles refer to those whose positions and shapes remain unchanged during the path-planning process. Therefore, the topography of submarine mountains can be regarded as a kind of broad static obstacle. In addition to mountains, the underwater environment also features natural obstacles such as reefs and functional obstacles for detecting sonar. Therefore, in this study, the threat areas of obstacles such as reefs are modeled and processed, and their shapes are approximately cuboids. The threat areas formed by monitoring systems such as sonar are modeled, and their shapes are approximately cylinders. The mathematical expressions of the two threat areas are:

(3) $$O_{cyl}=\left\{\begin{array}{cc} H_{j}^{c} & if\sqrt{{\left(x-x_{j}^{c}\right)}^{2}+{\left(y-y_{j}^{c}\right)}^{2}}\leq \;R_{j}^{2}\\ H(x,y) & otherwise \end{array}\right.$$

where $O_{cyl}$ is the original terrain elevation function, $\left(x_{j}^{c},y_{j}^{c}\right)$ is the center coordinate of the cylindrical obstacle, $R_{j}$ is its radius, and $H_{j}^{c}$ is its height. The forced elevation within the cylindrical area is Hic, forming an impassable vertical obstacle.

(4) $$O_{rect}=\left\{\begin{array}{cc} H_{k}^{r} & \mathrm{if}\;x_{k}^{r}\leq x\leq x_{k}^{r}+L_{k}^{x}\;\mathrm{and}\;y_{k}^{r}\leq y\leq y_{k}^{r}+L_{k}^{y}\\ H(x,y) & otherwise \end{array}\right.$$

where $O_{rect}$ is the original terrain elevation function, $\left(x_{k}^{r},y_{k}^{r}\right)$ is the lower left corner coordinate of the cuboid obstacle, $L_{k}^{x}$ and $L_{k}^{y}$ are the length and width of the cuboid, respectively, and $H_{k}^{r}$ represents its height. Define the elevation within the rectangular area as a fixed value $H_{k}^{r}$ to simulate artificial obstacles such as buildings. The terrain model, mountain peak model, and static obstacle model of this paper are shown in Figure 1, respectively.

#### 2.2.2. Dynamic Obstacle Modeling

Unlike static obstacles, dynamic obstacles refer to those whose positions and states change over time during the execution of tasks by the AUV. Common representatives include natural dynamic disorders dominated by fish schools and functional dynamic disorders dominated by unmanned ships. Therefore, although the modeling and obstacle avoidance strategies of dynamic obstacles are more challenging, they are also more in line with reality and have extremely high practical application significance. In most task scenarios, dynamic obstacles have a greater impact on path planning than static obstacles.

Just as the movement of fish schools follows the principles of fluid mechanics and self-organization [35], and the trajectories of unmanned ships are influenced by the wave spectrum model [36]. The motion of dynamic obstacles follows Newton’s second law F=ma, and its acceleration is composed of three parts:

a. Tendency term $\kappa \cdot \frac{p_{i}^{end}-p_{i}\left(t\right)}{∥p_{i}^{end}-p_{i}\left(t\right)∥}$: Simulates goal-oriented behavior, κ is the gain coefficient trend.

b. Damping term $\gamma \cdot v_{i}\left(t\right)$: characterization of fluid resistance ($\gamma =\frac{1}{2}C_{d}\rho A_{v}$,$C_{d}$ is the coefficient of resistance.)

c. Disturbance term $\xi_{a}\left(t\right)$: Environmental random perturbation ($\xi_{a}∼N\left(0,\Sigma \right)$, Σ=diag(0.2,0.2,0.1))

This model can accurately describe the characteristics, such as the fish school’s feeding behavior (κ term) and the influence of wave surges on unmanned vessels ($\xi_{a}$ term).

The dynamic obstacles in the environment are uncertain and time-varying, which puts forward higher requirements for the real-time perception, rapid planning, and dynamic adjustment of path planning. The dynamic obstacle model in this paper incorporates acceleration and random perturbations. The specific formula is as follows:

(5) $$R_{obs}=C$$

where $R_{obs}$ is the parameter shared by all dynamic obstacles, representing the minimum safe distance from the AUV after defining the dynamic obstacle as a sphere with a fixed collision radius. After that, define the motion equation of the dynamic obstacle. The position formula is as follows:

(6) $$P_{i}^{init}=\left(x_{i}^{init},y_{i}^{init},z_{i}^{init}\right)$$

(7) $$P_{i}^{goal}=\left(x_{i}^{goal},y_{i}^{goal},z_{i}^{goal}\right)$$

where $P_{i}^{init}$ and $P_{i}^{goal}$, respectively, represent the initial position and target position of the ith obstacle.

The dynamic obstacle model in this paper is a dynamic obstacle model that incorporates acceleration and random disturbances. To simulate the non-uniform motion characteristics of dynamic obstacles, the acceleration is defined as follows:

(8) $$a_{i}\left(t\right)=\kappa \cdot \frac{p_{i}^{\mathrm{end}}-p_{i}\left(t\right)}{∥p_{i}^{\mathrm{end}}-p_{i}\left(t\right)∥}-\gamma \cdot v_{i}\left(t\right)+\xi_{a}\left(t\right)$$

where $a_{i}$ is the tendency term, driving the obstacle to move towards the target point, and κ is the tendency gain coefficient; γ is the damping coefficient; $\xi_{a}$ is the random disturbance term, which follows a Gaussian distribution with a mean of zero and a covariance matrix of ∑a, and is used to simulate environmental disturbances. Then, define the velocity update equation. Let the speed adjust dynamically with the acceleration and superimpose random noise. The formula is as follows:

(9) $$v_{i}\left(t+\Delta t\right)=v_{i}\left(t\right)+a_{i}\left(t\right)\cdot \Delta t+\sigma_{v}\cdot N_{v}\left(t\right)$$

where $\sigma_{v}$ is the amplitude vector of velocity perturbation, and $N_{v}$ is the random variable of the standard normal distribution. Afterwards, the position is updated jointly by velocity and acceleration, and a position perturbation is introduced. The position update equation is as follows:

(10) $$p_{i}\left(t+\Delta t\right)=p_{i}\left(t\right)+v_{i}\left(t\right)\cdot \Delta t+\frac{1}{2}a_{i}\left(t\right)\cdot {\left(\Delta t\right)}^{2}+\sigma_{p}\cdot N_{p}\left(t\right)$$

where $p_{i}$ represents the position at time *t*, $v_{i}$ represents the velocity at time *t*, Δt represents the time step, $a_{i}$ represents the acceleration at time *t*, $\sigma_{p}$ represents the standard deviation of the position disturbance, and $N_{p}$ represents the random noise term related to the position.

Then, add a motion constraint condition to limit the height of the obstacle to ensure that it does not touch the seabed terrain. The safe depth threshold $\Delta h_{safe}$ is the threshold for the safe descent depth of the AUV. These constraint conditions also ensure consistency with the modeling of static obstacles, allowing dynamic obstacles to exist reasonably in three-dimensional space.

(11) $$z_{i}\left(t\right)\geq H_{base}\left(x_{i}\left(t\right),y_{i}\left(t\right)\right)+\Delta h_{safe}$$

where $H_{base}$ is the terrain elevation function, and $\Delta h_{safe}$ is the safe height margin.

### 2.3. Ocean Current Model with Complex Vortices

**Figure 2:**
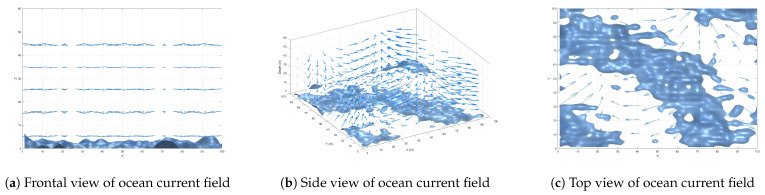
Ocean current field model.

In the actual navigation of AUVs, due to the limitations of the equipment itself, there are extremely high requirements for the energy consumption and navigation trajectory of underwater vehicles. Since the actual underwater environment is not constant, ocean currents and vortices fill it, resulting in an increase in the energy consumption of the AUV and an increase in trajectory deviation. In order to be closer to the actual situation, the factor of ocean currents should be considered in the path planning. In underwater AUV path planning, the ocean current field is usually three-dimensional and can exhibit different velocity and flow direction characteristics at different depths. To meet the requirements of hierarchical modeling, we can stratify the flow of the fluid, that is, apply the Navier–Stokes equation, respectively, on different water layers. In this paper, the following Navier–Stokes equation is introduced to model the underwater ocean current field [37]:

(12) $$\begin{array}{cc} \rho \left(\frac{\partial u_{i}\left(x,y,z\right)}{\partial t}\right) & +\rho \left(u_{i}\left(x,y,z\right)\cdot \nabla u_{i}\left(x,y,z\right)\right)\\ \; & =-\nabla p_{i}\left(x,y,z\right)+\mu \nabla^{2}u_{i}\left(x,y,z\right) \end{array}$$

where $\rho_{i}$ is the fluid density of the i-th layer, $u_{i}$ is the vector of the fluid velocity field of the i-th layer, $p_{i}$ is the fluid pressure field of the i-th layer, $\mu_{i}$ is the dynamic viscosity of the fluid in the i-th layer, and $\nabla p_{i}$ is the pressure gradient of the i-th layer, reflecting the change in fluid pressure. $\nabla^{2}u_{i}$ is the viscosity term of the i-th layer, which describes the viscous effect within the fluid. The specific modeling of the field of the ocean current is shown in Figure 2.

## 3. Improved Multi-Objective Particle Swarm Optimization Algorithm

The complex marine environment described in the second part—including irregular seabed topography, dynamic obstacles with uncertain trajectories, variously distributed ocean currents, and the need for coordination among multiple autonomous underwater vehicles—poses significant challenges to the traditional particle swarm optimization algorithm. These challenges include premature convergence in rugged terrain, insufficient response to moving threats, a decline in community diversity under current interference, and an inability to maintain a safe distance between multiple AUVs. To overcome these limitations, the improved multi-particle swarm optimization algorithm in this paper introduces three key innovations: the CPB initialization strategy that combines Chebyshev chaotic mapping, pre-iteration transient elimination, and boundary particle injection enhances population diversity to avoid local optima, and this strategy significantly improves the search efficiency and solution quality of the autonomous underwater vehicle path-planning task through enhancing the diversity and coverage of the initial population; the dynamic parameter adaptive mechanism balances exploration and exploitation under current interference; the hybrid Levy-differential perturbation can avoid dynamic obstacles in real time, and through ARIMA-guided particle injection ensure coordinated safety.

### 3.1. The Initialization Method of Hybrid Particle Swarm Based on CPB Method

#### 3.1.1. Hybrid Chaotic Initialization

In the path-planning application of the particle swarm optimization algorithm, the initialization strategy of the particle swarm directly affects the search performance, convergence speed, and the quality of the solution of the algorithm [38]. At present, in the initialization process of the particle swarm optimization algorithm, strategies such as random sampling, linear interpolation, and Latin hypercube sampling are commonly adopted. These traditional methods have high computational efficiency, are easy to implement, and are suitable for general low-dimensional optimization scenarios. However, in the underwater dynamic path-planning task of multi-AUV systems, these initialization methods have exposed several deficiencies.

In recent years, chaotic mapping, as an effective improvement method of swarm intelligence algorithms, has been widely applied in the initialization stage of optimization algorithms due to its advantages, such as strong ergodic ability, good pseudo-randomness, and high initial value sensitivity. Commonly used chaotic mappings include the Logistic mapping, Tent mapping, sine mapping, etc. However, these traditional chaotic mapping models still face some challenges in the dynamic path-planning task [39]. On the one hand, the sequences they generate have limited coverage ability in the boundary region of the search space, resulting in insufficient exploration of edge paths by the particle swarm during the dynamic obstacle avoidance process. On the other hand, these mapping models have fixed control parameters and insufficient adaptability, making it difficult to respond dynamically to complex environmental changes.

Therefore, this paper proposes a strategy for initializing the improved Chebyshev chaotic map using the dynamic order adjustment mechanism and the boundary enhancement operator. The iterative formula of the traditional Chebyshev mapping is as follows:

(13) $$x_{k+1}=\cos\left(n\cdot \arccos(x_{k})\right),\;x_{k}\in \left[-1,1\right]$$

The traditional research on chaotic mapping mainly focuses on static optimization problems. Although the chaotic sequence it generates has ergodicity, in dynamic path planning, there are still problems such as the N-order fixation (usually taking 2 or 4) in traditional methods, which makes it difficult to adapt to dynamic obstacles and ocean current disturbances. There are problems, such as insufficient boundary sensitivity in multi-AUV cooperative obstacle avoidance, which cannot meet the requirements of edge path detection. Therefore, this paper first adopts the dynamic order adjustment mechanism to adaptively adjust the order *n* according to the environmental complexity. The formula is as follows:

(14) $$n=2+\lfloor 3\cdot \frac{\rho_{max}}{\rho_{obs}}\rfloor$$

where $\rho_{obs}$ is the real-time obstacle density, and $\rho_{max}$ is the preset maximum obstacle density. It effectively addresses the problems of excessive randomization and decreased convergence speed caused by using high chaotic intensity in the traditional fixed-order Chebyshev mapping in dynamic underwater scenes, as well as the insufficient diversity and increased obstacle avoidance failure rate caused by the use of low chaotic intensity. Through the dynamic switching of order *n*, the algorithm exhibits differentiated search behaviors in different regions. In the obstacle area, local disturbances have been enhanced, the ability to evade local optima has been improved, and the potential for obstacle avoidance has been strengthened. In the sparse area of obstacles, the chaotic intensity is reduced, a smooth sequence is generated, the global optimum is rapidly approached, and convergence is accelerated.

#### 3.1.2. Pre-Iterative Transient Elimination

In the initial iteration stage of chaotic mapping, there will be a non-equilibrium state before the system transitions from the initial value to the steady-state chaos, that is, the transient phenomenon. At this stage, the sequence has problems such as insufficient coverage of the solution space by the sequence, resulting in insufficient ergodicity and strong correlation between adjacent values, and destroying pseudo-randomness. Therefore, this paper proposes a pre-iterative elimination mechanism. Its formula is as follows:

(15) $$\left\{\begin{array}{cc} x_{0} & ∼U(-1,1)\\ x_{pre}\left(m\right) & =f\left(x_{pre}\left(m-1\right)\right),\;m=1,2,\ldots ,M_{pre}\\ X_{i} & =\phi (x_{pre}\left(M_{pre}\right)) \end{array}\right.$$

where $x_{0}$ represents the initial value of the chaotic mapping, which is a uniformly randomly generated vector within the interval of [−1, 1]. $x_{pre}$ is the chaotic value after pre-iteration. $X_{i}$ represents the position vector of the i-th particle in the solution space. $M_{pre}$ represents the number of pre-iterations, that is, the number of iterations for performing the chaotic mapping before officially initializing the particle swarm using the chaotic sequence. This process can eliminate the transient effect by transitioning the chaotic system from the initial value to the steady-state chaotic state through a sufficient number of pre-iterations, making the steady-state chaotic sequence have higher randomness and ergodicity, and improving the sequence quality [40]. Among them, the expression of ϕ is

(16) $$\phi \left(x\right)=\frac{x+1}{2}\cdot \left(ub-lb\right)+lb$$

where lb represents the lower bound of each dimension in the solution space, that is, the minimum value of the optimization variable. ub represents the upper bound of each dimension in the solution space, that is, the maximum value of the optimization variable. This formula defines the search space for the feasible range of particle positions. In addition, map the chaotic sequence to the actual solution space.

#### 3.1.3. Boundary Particle Injection

The traditional PSO algorithm relies on randomly generating particles, and the probability of particle generation in the boundary region of the solution space is extremely low. Therefore, injecting boundary particles can prevent the algorithm from ignoring these regions due to the insufficient randomness of the initial population. However, in high-dimensional or complex constraint problems, the optimal solution may be located at the boundary of the solution space. Meanwhile, although Chebyshev mapping generates uniform sequences, under high-dimensional or dynamic constraints, boundary coverage may still be insufficient [41]. Therefore, this paper proposes a boundary particle injection strategy that dynamically adjusts the positions and quantities of injected particles according to real-time environmental changes. Let the characteristic vector of the environmental state at time *t* be the following formula

(17) $$E\left(t\right)={\left[\begin{array}{c} \rho_{obs}\left(t\right),\;v_{current}\left(t\right),\;H_{map}\left(t\right) \end{array}\right]}^{T}$$

where *E* represents the characteristic vector of the environmental state at time *t*, $\rho_{obs}$ represents the density of obstacles, $v_{current}$ represents the ocean current velocity field, and $H_{map}$ represents the dynamic terrain elevation. Then define the number of injected boundary particles as follows:

(18) $$N_{boundary}\left(t\right)=N_{base}+\left\lfloor k\cdot \frac{\rho_{max}}{\rho_{obs}\left(t\right)}\right\rfloor$$

where $N_{boundary}$ represents the number of injected particles, $N_{base}$ represents the number of fundamental particles with fixed upper and lower bound vertices, *k* is the sensitivity coefficient, and $\rho_{max}$ is the preset maximum obstacle density threshold. This step can inject more particles to enhance the edge exploration ability when obstacles are denser. Next, the Voronoi diagram is constructed, and the formula is as follows.

(19) $$V\left(o_{i}\right)=\left\{x\in R^{2}\;|\;∥x-o_{i}∥\leq ∥x-o_{j}∥,\;\forall j\neq i\right\}$$

where both $o_{i}$ and $o_{j}$ are in the set of obstacle positions $O=\{o_{1},o_{2},\ldots ,o_{M}\}$. Furthermore, the edge set E represents the boundary line between adjacent Voronoi regions. Then, for each edge e∈E, calculate the minimum width. The formula is as follows:

(20) $$W\left(e\right)=\min_{x\in e}\left(∥x-o_{i}∥+∥x-o_{j}∥\right),\;o_{i},o_{j}\in O$$

Then, the key channel screening is carried out. The candidate edge set of the injected particles is represented by the following formula:

(21) $$E_{critical}=\left\{e\in E\;|\;W_{safe}\leq W\left(e\right)\leq 2W_{safe}\right\}$$

where $E_{critical}$ represents the candidate edge set of the injected particles, and $W_{safe}$ represents the safe passage width of the AUV. Then, highly dynamic constraints are used to adjust the vertical coordinate $z_{i}$ according to the real-time terrain. The formula is as follows:

(22) $$z_{i}=\max\left(H_{map}\left(x_{i},y_{i}\right)+\Delta h_{min},\;\min\left(z_{raw},\;H_{map}\left(x_{i},y_{i}\right)+\Delta h_{max}\right)\right)$$

where $z_{raw}$ is the height value generated by the original chaotic mapping, and $\Delta h_{min}$ and $\Delta h_{max}$ are the safe height ranges. Then, dynamic adjustment and optimization are carried out, using the prediction-correction mechanism. First, the autoregressive integral moving average model is used to predict the environmental state of the future $T_{p}$ step, and its formula is as follows:

(23) $$\hat{E}\left(t+T_{p}\right)=\sum_{i=1}^{p}\phi_{i}\;E\left(t-i\right)+\sum_{j=1}^{q}\theta_{j}\;\epsilon \left(t-j\right)+\epsilon \left(t\right)$$

where $\phi_{i}$ and $\theta_{j}$ represent the model parameters, and ϵ is the white noise element. *p* and *q* represent the order of the autoregressive and moving average, respectively. *E* is the historical value of the time series. Next, the correction of the position of the particles is carried out, and the predicted values are corrected based on the actual observed values. The formula is as follows.

(24) $$x_{i}\left(t\right)=\alpha \;{\hat{x}}_{i}\left(t\right)+\left(1-\alpha \right)\;x_{i}^{obs},\;\alpha \in \left[0,1\right]$$

where $x_{i}$ represents the predicted position, $x_{i}^{obs}$ represents the actual observed position, and α represents the control prediction trust degree. Finally, the multi-objective scoring function is defined as:

(25) $$S\left(x_{i}\right)=w_{1}\cdot \frac{1}{W(x_{i})}+w_{2}\cdot \rho_{obs}\left(x_{i}\right)+w_{3}\cdot ∥v_{current}\left(x_{i}\right)∥$$

where *S* is the critical score of particle $x_{i}$, *W* represents the width of the channel where it is located, and $w_{1}$, $w_{2}$, $w_{3}$ are the weight coefficients, which satisfy the sum of 1. Finally, the weight is adaptively adjusted to dynamically optimize the weight based on the performance of the historical path. The formula is as follows:

(26) $$w_{j}\left(t+1\right)=w_{j}\left(t\right)+\eta \cdot \frac{\partial J}{\partial w_{j}}$$

where η represents the learning rate and *J* represents the path quality index. Its value is equal to the weighted sum of length, energy consumption, and safety margin.

### 3.2. Dynamic Adaptive Parameter Adjustment

The traditional PSO algorithm adopts a fixed parameter configuration. However, fixed static parameters cannot meet the requirements of different stages of the search process, which is prone to problems such as slow convergence in the early stage or oscillation in the later stage, and difficulty in dealing with complex marine environments, such as dynamic obstacles and time-varying ocean currents. Therefore, the inertial weight exponential attenuation mechanism is introduced, and its formula is as follows:

(27) $$\omega \left(t\right)=0.4+0.5\cdot e^{-\frac{3t}{T_{max}}}$$

where ω is the inertia weight and $T_{max}$ is the maximum number of iterations. When t < 0.2$T_{max}$, it is the initial stage. The particles maintain high-speed movement and enhance the global exploration ability. When 0.2$T_{max}$ < t < 0.7$T_{max}$, it is the transitional stage, balancing exploration and development. When 0.7$T_{max}$ < t, it is the later stage, allowing the particles to accelerate and converge to the optimal solution. Then, the asymmetric learning factor design is used, and its formula is as follows:

(28) $$\left\{\begin{array}{cc} c_{1}\left(t\right) & =2.0-1.5\cdot \frac{t}{T_{max}}\\ c_{2}\left(t\right) & =0.5+1.5\cdot \frac{t}{T_{max}} \end{array}\right.$$

where $c_{1}$ is the individual learning factor and $c_{2}$ is the social learning factor. When C1>C2, it is the period dominated by individual cognition, and particles are encouraged to explore the historical optimal region of the individual. When C1<C2, it is the period dominated by social cognition, promoting the information sharing of particle groups. This method can effectively increase the convergence speed and improve the convergence efficiency.

### 3.3. Hybrid Perturbation Strategy

In order to effectively reduce the problems such as premature convergence, diversity attenuation, and dynamic response lag of traditional PSO. This paper proposes a hybrid perturbation strategy combining Levy flight and differential variation.

#### 3.3.1. Levy Flight Disturbance

The Levy flight perturbation is used as the dominant factor for global exploration, and its formula is as follows:

(29) $$\Delta x=0.01\cdot \frac{u}{{\left|v\right|}^{1/\beta }}$$

where Δx is the perturbation step-size vector, β is the Levy index, which is generally taken as 1.5, and *u* and *v* are random number generators. Its expression is as follows:

(30) $$\left\{\begin{array}{cc} u & ∼N(0,1.5)\\ v & ∼N(0,1) \end{array}\right.$$

#### 3.3.2. Differential Mutation Strategy

In order to solve the problems of particle aggregation in the later stage of iteration, which causes the entropy value to drop to the danger zone, and the fact that traditional PSO is prone to stagnation in flat terrain and lacks gradient information, this paper introduces the differential mutation strategy, and its mutation formula is as follows:

(31) $$x_{mut}=g_{best}+0.1\cdot \left(x_{r1}-x_{r2}\right)$$

where $x_{mut}$ is the new position after mutation, $g_{best}$ is the global historical best position, $x_{r1}$ and $x_{r2}$ are randomly selected particle positions, and it is forced that r1≠r2≠i to avoid invalid differences.

## 4. Improved Dynamic Window Approach

### 4.1. Adaptive Dynamic Window Generation

Traditional DWA adopts a fixed-resolution velocity window. Therefore, in complex scenarios, there will be problems such as insufficient trajectory coverage in obstacle-dense areas or redundant calculations due to the inability of the fixed resolution to capture the feasible paths of narrow channels. Therefore, this paper adopts the strategy of dynamically adjusting the angular velocity resolution, and its formula is as follows:

(32) $$\Delta \omega \left(t\right)=\Delta \omega_{base}\cdot \left(1+\frac{\rho_{obs}\left(t\right)}{\rho_{\max}}\right)$$

where $\omega_{base}$ represents the basic resolution, $\rho_{obs}$ represents the local obstacle density, and $\rho_{max}$ represents the density threshold. This formula can dynamically adjust the environmental perception resolution, improve the path discovery rate in complex areas, and optimize the computational efficiency, reducing trajectory evaluations in simple scenarios.

### 4.2. Multi-Objective Optimization Cost Function

The traditional dynamic window method usually only optimizes the target proximity and obstacle avoidance distance. However, in complex scenarios, the failure to consider motion smoothness may lead to path jitter. The optimization of a single target is prone to falling into suboptimal solutions and cannot globally balance the multi-dimensional performance. Therefore, a single goal or a few goals cannot meet the actual needs. This paper introduces a multi-objective cost function that comprehensively evaluates trajectory safety, efficiency, smoothness, and energy consumption:

(33) $$C_{total}=\sum_{j=1}^{4}w_{j}\left(t\right)\;C_{j}$$

where $C_{total}$ represents the total cost function, which is used to evaluate the global advantages and disadvantages of candidate trajectories, enabling the algorithm to balance safety, efficiency, smoothness, and energy consumption when choosing paths. $w_{j}$ represents the time-varying weight of the jth term. $C_{j}$ represents the jth sub-cost item, corresponding to four optimization objectives. $C_{1}$ stands for $C_{goal}$, representing the closeness to the goal. $C_{2}$ stands for $C_{obs}$, representing the obstacle avoidance distance. $C_{3}$ stands for $C_{smooth}$, representing the smoothness of motion. $C_{4}$ stands for $C_{energy}$, representing energy consumption. The respective formulas are as follows:

(34) $$C_{goal}=1-\frac{\pi }{|\theta_{AUV}-\theta_{goal}|}$$

where $\theta_{AUV}$ represents the heading angle in the current row of AUV, and $\theta_{goal}$ represents the target direction angle.

(35) $$C_{obs}=\frac{1}{d_{min}+\epsilon }$$

where $d_{min}$ represents the nearest distance to the object, and ϵ takes a very small value. Its function is to prevent the denominator from being 0.

(36) $$C_{smooth}=\frac{1}{N-1}\sum_{i=1}^{N-1}\left|\theta_{i+1}-\theta_{i}\right|$$

where $\theta_{i}$ is the heading Angle of trajectory segment *i*, and *N* is the number of trajectory points.

(37) $$C_{energy}=\int_{0}^{T}\left(k_{1}v^{2}+k_{2}\omega^{2}\right)\;dt$$

where $k_{1}$ and $k_{2}$ are energy consumption coefficients, and *v* and ω are linear velocities and angular velocities, respectively. Then, the weight adaptive mechanism is utilized, and its formula is as follows:

(38) $$w_{j}\left(t\right)=\frac{e^{-\lambda_{j}t}}{\sum_{k=1}^{4}e^{-\lambda_{k}t}},\;\lambda =\left[0.5,\;1.0,\;0.3,\;0.7\right]$$

where $\lambda_{j}$ represents the attenuation coefficient of the jth term, which controls the rate at which the weight changes over time. *t* represents the current time step or the number of iterations. This enables the high weight in the initial stage to focus on quickly approaching the target and avoiding obstacles, while the high weight in the later stage focuses on optimizing smoothness and energy consumption.

### 4.3. Trajectory Prediction and Correction Mechanism

In underwater environments with dynamic obstacles (e.g., moving fish schools), traditional DWA may fail to avoid collisions due to a lack of trajectory prediction. This paper incorporates motion prediction to forecast obstacle movements and correct planned trajectories accordingly [42]. For each predicted time step, dynamic obstacles are extrapolated using constant velocity or learning-based motion models. Future conflicts are detected through forward simulation, and the candidate trajectories are corrected via penalty adjustments to the cost function. This significantly improves DWA performance in dynamic, cluttered environments. Its formula is as follows:

(39) $${\hat{v}}_{obs}\left(t+\Delta t\right)=\sum_{i=1}^{p}\phi_{i}v_{obs}\left(t-i\right)+\sum_{j=1}^{q}\theta_{j}\epsilon \left(t-j\right)+\epsilon \left(t\right)$$

where ${\hat{v}}_{obs}$ is the predicted velocity of the obstacle at time t+Δt, *p* is the autoregressive order, $\phi_{i}$ is the autoregressive coefficient, *q* is the moving average order, $\theta_{j}$ is the moving average coefficient, and ϵ is the white noise term. When the predicted collision probability is greater than 50%. The ARIMA prediction model synergizes with the physics-based acceleration model. This hybrid approach leverages both physical priors and data-driven correction. In addition, the trajectory correction rule is used, and its formula is as follows:

(40) $$v_{new}=v_{prev}\cdot \left(1-\frac{d_{safe}}{∥{\hat{p}}_{obs}-p_{AUV}∥}\right)$$

where $v_{prev}$ represents the current linear velocity of the robot, $p_{obs}$ is the predicted position of the obstacle, $p_{AUV}$ is the current position of AUV, and $d_{safe}$ is the dynamic safety distance. The formulas are as follows:

(41) $$d_{safe}=v_{AUV}\cdot t_{resp}+\delta_{min}$$

where $t_{resp}$ is the system response time, and $\delta_{min}$ is the minimum static safety margin. The above strategies can adjust the speed according to the approaching rate of obstacles to avoid sudden braking. The dynamic safety distance is adaptively adjusted with the AUV speed, and a larger buffer space is reserved at high speeds.

## 5. Multi-AUV Cooperative Path Planning Based on Improved PSO-DWA

### 5.1. Multi-Stage Planning Framework Design

This paper proposes a two-tier architecture of “static global planning—dynamic local adjustment”, and realizes the collaboration of multiple AUVs through the path segmentation strategy [43]. Therefore, the set of global path segmentation points is defined as follows:

(42) $$P_{s}=\left\{p_{k}\;|\;p_{k}=p_{gb}+\lambda_{k}\left(p_{end}-p_{gb}\right),\;\lambda_{k}\in \left(0,1\right)\right\}$$

where $P_{s}$ is the set of global path segmentation points; $p_{k}$ is the k-th segmentation point, which is used to trigger the critical position of local dynamic programming; $p_{gb}$ is the global optimal path point; $p_{end}$ is the destination of the goal; $\lambda_{k}$ is the dynamic segmentation coefficient, which is used to control the relative position of the segmentation point and the global optimal path point. Its formula is as follows:

(43) $$\lambda_{k}=1-\frac{∥v_{c}\left(p_{k}\right)∥}{v_{c_{max}}}+\alpha \cdot \frac{\rho_{obs}\left(p_{k}\right)}{\rho_{max}}$$

where $v_{c}\left(p_{k}\right)$ is the ocean current velocity vector at position $p_{k}$. $v_{c_{max}}$ is the speed of the maximum ocean current; $\rho_{obs}\left(p_{k}\right)$ is the local obstacle density at position $p_{k}$; $p_{max}$ is the threshold of the maximum obstacle density; α is the obstacle sensitive factor, which is used to adjust the influence weight of the obstacle density on the segmentation coefficient.

The collaborative triggering mechanism monitors the AUV spacing in real time through the following formula:

(44) $$\Delta p_{ij}=∥p_{i}-p_{j}∥$$

where $\Delta p_{ij}$ is the Euclidean distance between AUVs, representing the real-time spatial distance between the i-th and j-th AUVs. When $d_{safe}<\Delta p_{ij}<2d_{safe}$, the collaborative constraints are activated to avoid the frequent start-stop problem caused by the traditional fixed threshold.

### 5.2. Reconstruction of Multi-Objective Optimization Functions

This paper proposes a four-dimensional time-varying weight objective function, adding communication topology and task priority constraints.

(45) $$F_{total}=\sum_{i=1}^{4}w_{i}\left(t\right)f_{i}+\mu \left(g_{1}+g_{2}\right)$$

where $F_{total}$ is the comprehensive objective function; $w_{i}$ is the time-varying weight coefficient; $f_{i}$ is the sub-objective function; μ is the constraint penalty weight, which is used to balance the violation degree of the objective function and the constraint conditions; $g_{1}$ is the communication topology constraint; $g_{2}$ is the task timeliness constraint. The formulas of $g_{1}$, $g_{2}$, and each sub-objective function are as follows:

(46) $$g_{1}=\sum_{(i,j)\in \epsilon }\max\left(0,\;d_{com}-∥p_{i}-p_{j}∥\right)$$

where ϵ is the set of communication topology edges, describing the communication connection relationship between AUVs; $d_{com}$ is the maximum communication distance.

(47) $$g_{2}=\sum_{i=1}^{M}∥t_{i}-{\hat{t}}_{i}∥$$

where $t_{i}$ is the actual arrival time of the i-th AUV; ${\hat{t}}_{i}$ is the task requirement time for the i-th AUV.

(48) $$f_{1}=\sum_{j=1}^{N}\left[\frac{d_{obs}\left(p_{j}\right)}{1+h_{safe}\cdot \Delta h\left(p_{j}\right)}\right]$$

$f_{1}$ is the path safety degree function; $d_{obs}$ is the Euclidean distance from the path point $p_{j}$ to the nearest point nearest obstacle. $\Delta h\left(p_{j}\right)=\left|h\left(p_{j}\right)-H_{base}\left(p_{j}\right)\right|$, that is, the height deviation of the path point $p_{j}$.

(49) $$f_{2}=\sum_{i\neq j}\max{\left(0,\frac{d_{safe}-∥p_{i}-p_{j}∥}{d_{safe}}\right)}^{2}$$

$f_{2}$ is the cooperative separation function. When the AUV spacing is less than the safety spacing of multiple AUVs, the function value increases squared as the distance decreases, forcibly maintaining the formation safety.

(50) $$f_{3}=\sum_{j=1}^{N}\left[\alpha {∥v_{j}∥}^{2}+\beta {∥a_{j}∥}^{2}+\gamma ∥v_{j}-v_{c}\left(p_{j}\right)∥\right]$$

$f_{3}$ is the energy loss degree function, and $v_{j}$, $a_{j}$, $v_{c}$ are, respectively, the velocity, acceleration, and ocean current velocity of AUV at the path point $p_{j}$. α,β,γ are the weight coefficients.

(51) $$f_{4}=\sum_{j=2}^{N-1}\arccos\left(\frac{\Delta p_{j}\cdot \Delta p_{j+1}}{∥\Delta p_{j}∥∥\Delta p_{j+1}∥}\right)$$

$f_{4}$ is the path smoothness function. The formula $\Delta p_{j}=p_{j}-p_{j-1}$ is the vector difference from the path segment $p_{j}$ to $p_{j-1}$. The formula $\Delta p_{j+1}=p_{j+1}-p_{j}$ represents the vector difference from the path segment $p_{j+1}$ to $p_{j}$. arccos is used to calculate the angle between two vectors. By minimizing the total included angle sum, ensure a smooth path, and reduce unnecessary turns.

The time-varying weight strategy can focus on path security in the early stage of iteration and optimize energy consumption in the later stage. Communication constraints can ensure that multiple AUVs maintain a communication distance greater than the safe distance in complex terrains.

### 5.3. Collaborative Path Smoothing Enhancement

In order to enhance the security of multi-machine collaboration, optimize the smoothness of motion, and improve environmental adaptability, this paper uses the collaborative path smoothing enhancement strategy, and the formula is as follows:

(52) $$\left\{\begin{array}{cc} Q_{i}\left(t\right) & =\sum_{k=0}^{n}N_{k,3}\left(t\right)\;P_{k}^{\left(i\right)}\\ ∥Q_{i}\left(t\right)-Q_{j}\left(t\right)∥ & \geq d_{safe}+\eta \cdot v_{rel}\\ \frac{d^{3}Q_{i}}{dt^{3}} & \leq J_{max} \end{array}\right.$$

where $Q_{i}$ represents the B-spline path function of the i-th AUV; $N_{k,3}$ is the cubic B-spline basis function; $P_{k}^{\left(i\right)}$ represents the set of control points of the i-th AUV; $v_{rel}=∥v_{i}-v_{j}∥$, which is the relative velocity between AUV; η is the speed-sensitive factor, regulating the influence of speed on the safety margin; $J_{max}$ is the maximum jerk, and is used to limit the smoothness of the path.

By enhancing the safety margin and collision-avoidance success rate in narrow passages and using a parallel solution strategy, the computational time consumption is significantly reduced.

### 5.4. Dynamic Window Collaborative Expansion

The stand-alone DWA only considers static obstacles and its own motion constraints. Therefore, a new collaborative constraint term is added. Through cooperative AUV window constraint collision-avoidance maneuvers, a chain reaction can be triggered, with a mandatory reserved collaborative response time to avoid conflicting decisions. The constraint formula of the multi-AUV cooperative window is as follows:

(53) $$W_{d}^{\left(i\right)}=\{\left(v,\omega \right)\;|\;\left\{\begin{array}{c} \frac{a_{max}}{∥v_{j}-v_{i}∥}\leq \Delta t\leq t_{resp}\\ ∠\left(v_{i},v_{j}\right)\geq \theta_{safe} \end{array}\right.$$

where $W_{d}^{\left(i\right)}$ is the set of dynamic Windows of the i-th AUV, including all feasible combinations of velocity and angular velocity; Δt is the prediction time domain of the dynamic window method; $t_{resp}$ is the system response time; $∠(v_{i},v_{j})$ is the velocity vector included Angle, and $\theta_{safe}$ is the dynamic safety included Angle. Its formula is as follows:

(54) $$\theta_{safe}=30^{{}^{\circ}}\cdot \left(1+\frac{\rho_{obs}}{\rho_{max}}\right)$$

Meanwhile, using the collaborative evaluation function and quantifying the key indicators of multi-machine collaboration, the collision-avoidance decision and motion coordination in path planning are optimized. The specific formula is as follows:

(55) $$C_{collab}=\sum_{j\in N_{i}}\left[\frac{1}{{∥p_{i}-p_{j}∥}^{2}}+\frac{∥v_{i}-v_{j}∥}{v_{max}}\right]$$

where $N_{i}$ is the neighbor set determined by the i-th AUV through communication topology or sensing range.

### 5.5. Computational Complexity and Scalability Analysis

Compared with conventional meta-heuristic frameworks, the proposed hybrid PSO-DWA architecture exhibits a modular yet interdependent computational pattern. Its complexity is not only determined by the iteration scale but also shaped by the adaptivity and multi-level feedback mechanisms introduced in this work.

Let *N* be the number of particles, *T* the number of iterations, *D* the path dimensionality (i.e., number of nodes per AUV), and *M* the number of AUVs. The global planning stage based on improved PSO integrates CPB initialization, adaptive parameters, and hybrid Levy-differential mutation. Each cost evaluation per particle involves collision checks, ocean current estimation, terrain safety margin computation, and multi-objective aggregation, leading to a cost of O(D) per particle. Consequently, the global planning complexity scales as:

(56) O(N·D·T)

However, the CPB initialization introduces a front-end overhead. Specifically, Chebyshev chaotic mapping with $M_{pre}$ pre-iterations, Voronoi-based boundary injection, and ARIMA-based prediction-correction require additional preprocessing time. Denoting $P_{inj}$ as the number of injected edge particles and $C_{env}$ as the complexity of terrain and current feature extraction, the extended initialization complexity becomes:

(57) $$O(M_{pre}\cdot N+P_{inj}\cdot C_{env})$$

This cost significantly enhances early-stage diversity and reduces the number of required iterations *T* for convergence.

During online execution, the DWA component is triggered adaptively based on current, obstacle density, and inter-AUV proximity. For each AUV, if the dynamic window samples *K* velocity pairs per step and executes *S* local adjustments along its trajectory, the local planning cost becomes:

(58) O(M·S·K)

Moreover, the proposed trajectory prediction module, which blends physics-informed acceleration with ARIMA-based velocity forecasting, introduces a constant-time correction step, not significantly impacting the overall per-step complexity.

In total, the hybrid algorithm’s complexity can be expressed as:

(59) $$O(N\cdot D\cdot T+M_{pre}\cdot N+P_{inj}\cdot C_{env}+M\cdot S\cdot K)$$

The design explicitly separates high-complexity global search and lightweight real-time local corrections. Furthermore, particle-level and AUV-level processes are parallelizable by design, enabling deployment on GPU-enabled simulation clusters or multi-core embedded processors. Importantly, the framework tolerates asynchrony in dynamic updates, which makes it robust under partial observability and variable latency scenarios.

Thus, although the algorithm introduces moderate computational overhead compared to traditional PSO or DWA, its multi-resolution planning architecture and bioinspired enhancements ensure scalable performance and real-time responsiveness in complex 3D marine environments with uncertain terrain and fluid dynamics.

### 5.6. Flowchart of Multi-AUVs Path Planning Based on IMOPSO Algorithm

This paper proposes a multi-AUV dynamic path-planning method based on the combination of the improved Multi-Objective Particle Swarm Optimization Algorithm and the Dynamic Window Algorithm. This method fully integrates the global optimization ability and the local obstacle avoidance ability, and is capable of quickly generating safe and feasible paths in a dynamic and complex environment. The overall process of this algorithm includes steps such as initialization, particle update, local dynamic optimization, path evaluation, and update. To present its execution logic more intuitively, the main process is now presented in the form of pseudo-code, as shown in Algorithm 1.

To clarify the criteria for determining the “non-convergence” condition, this paper adopts the following criteria: Firstly, the algorithm sets a maximum number of iterations $T_{max}$ as a forced stop condition to prevent excessive computation; Then, if the rate of change in the global optimal fitness for several consecutive generations is lower than the preset threshold ϵ, it is considered that the population tends to converge; Finally, if the variance of the particle’s position or velocity gradually stabilizes, that is, the fluctuation is less than the threshold δ, it can also be determined that the convergence state has been reached. The mathematical expression is as follows:

(60) $$\left|f_{t}^{best}-f_{t-K}^{best}\right|<\epsilon$$

(61) $$Var(x_{t})<\delta$$

where $f_{t}^{best}$ represents the global optimal fitness of the *t*-th generation, *K* is the number of consecutive observation generations, and $x_{t}$ represents the position vector of all particles in the *t*-th generation. In this paper, the empirical settings are $\epsilon =10^{-3}$, $\delta =10^{-4}$, and K=10. This criterion takes into account both the convergence of the solution and the diversity of the population, which helps to avoid premature convergence and simultaneously improves the computational efficiency.
**Algorithm 1:** Hybrid Global-Local Path Planning for Multi-AUV.
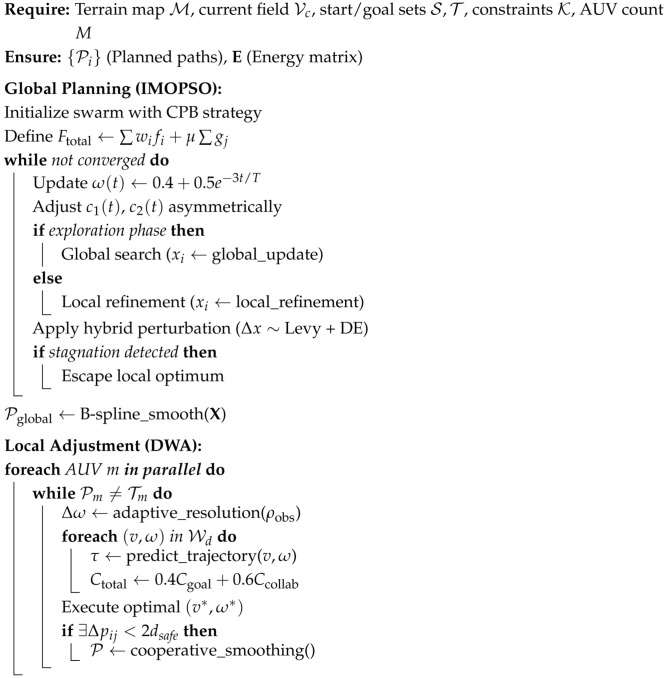


In order to verify the effectiveness of the improved strategy, this paper designs the initialization comparison experiment, the global path planning of each algorithm, and the dynamic cooperative path-planning experiment of multiple underwater robots. The effectiveness of the improved hybrid algorithm in the problem of underwater environment path planning and the feasibility of the improved hybrid algorithm were verified, respectively.

## 6. Simulation Result and Analysis

### 6.1. Initialization Comparison Experiment

In this paper, the particle swarm initialization strategy of the CPB strategy is used to improve the PSO algorithm. It includes the Chebyshev hybrid initialization, pre-iterative elimination, and boundary particle injection methods. Meanwhile, dynamic adaptive parameter adjustment and the addition of hybrid perturbation strategies are used. Therefore, each method is compared with the original PSO algorithm, respectively. The relevant parameters of IMOPSO are shown in Table 1.

**Figure 3:**
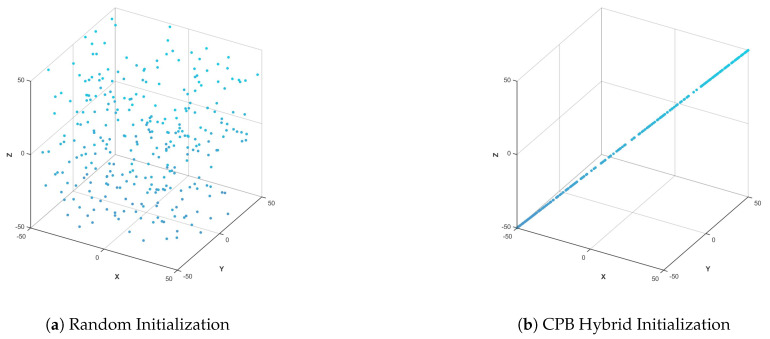
Initialization comparison.

Figure 3 shows the comparison between the random initialization of PSO and the mixed initialization of CPB in this paper. In Figure 3a, the particles are concentrated in the center of the search space, and the boundary area is sparse. In Figure 3b, the particles uniformly fill the entire space, and the edges are well covered. It can be seen that the initialization strategy adopted in this paper conducts a more comprehensive global search and is less likely to fall into a local optimum.

**Figure 4:**
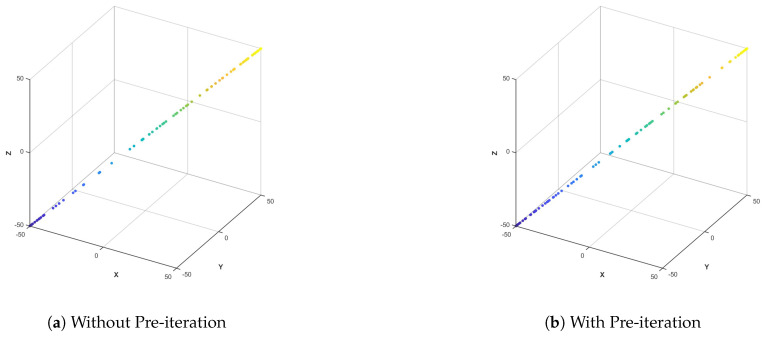
Comparison with and without pre-iteration.

Figure 4 shows the comparison of the PSO algorithm with and without pre-iteration. In Figure 4a, there is no pre-iteration, and fluctuations, offsets, and local clustering occur in the particle concentration. Figure 4b has pre-iteration, and the particle distribution is symmetrical, regular, and uniform. It can be seen that the pre-iteration strategy adopted in this paper can filter out unstable states and improve the initialization balance.

**Figure 5:**
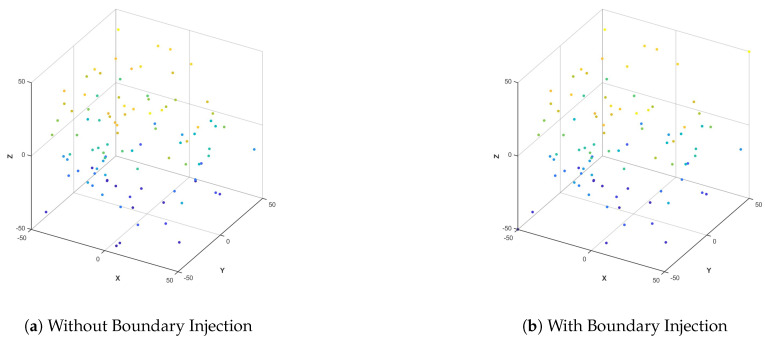
Comparison with and without boundary injection.

Figure 5 shows a comparison chart of the PSO algorithm with and without boundary particle injection. In Figure 5a, there is no boundary particle injection, and there are a large number of blanks in the edge area. In Figure 5b, there is boundary particle injection, and the boundary particles clearly fill the periphery. It can be seen that the boundary particle injection strategy adopted in this paper can improve the boundary search ability and avoid losing the optimal boundary solution.

**Figure 6:**
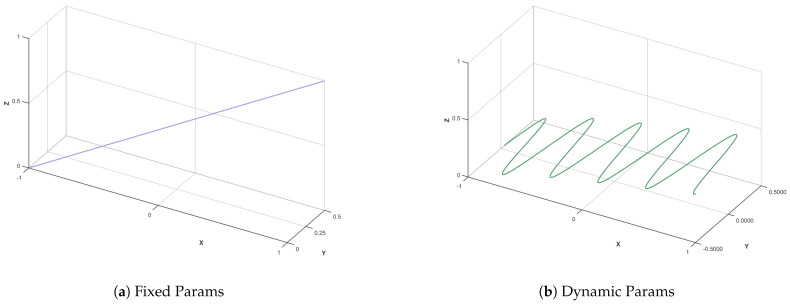
Dynamic parameter comparison.

Figure 6 shows the comparison of the PSO algorithm with and without dynamic parameter adjustment. In Figure 6a is a fixed parameter. The convergence path is straight or has large jitter, and it is prone to premature convergence. In Figure 6b shows dynamic parameters. The inertia weight + learning factor is adjusted over time, and the path gradually converges. It can be seen that the dynamic parameter adjustment strategy adopted in this paper can improve the convergence of the algorithm and the global search ability.

**Figure 7:**
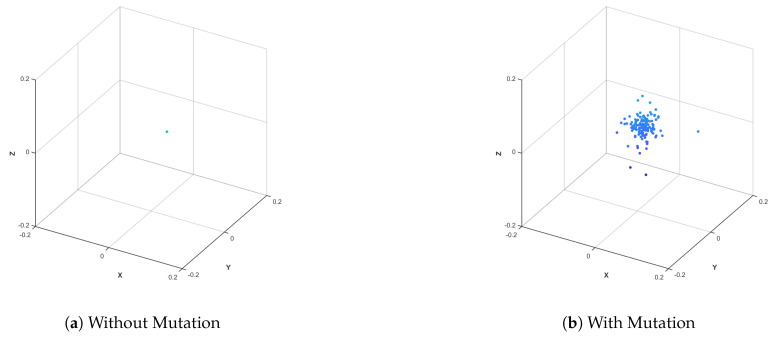
Comparison of mixed disturbances with and without.

Figure 7 shows the comparison of the PSO algorithm with and without a mixed perturbation strategy. In Figure 7a, there is no disturbance. All particles rigidly revolve around the global optimal point with almost no divergence. In Figure 7b features mixed perturbations, appropriate particle jumps/diffuses, and increases exploration potential. It can be seen that the hybrid perturbation strategy adopted in this paper does not disrupt convergence and can also escape from a local optimum.

### 6.2. Parameter Sensitivity Analysis

To quantitatively evaluate the impact of key parameters on algorithm performance, this section conducts sensitivity experiments on the individual learning factor $c_{1}$, the Levy flight step factor $\alpha_{Levy}$, and the inertia weight $w_{pso}$. The test environment is a three-dimensional marine scene, with a fixed population size of *N* = 100 and a maximum number of iterations $T_{max}$ = 50. The rest of the parameters remain unchanged. Table 2 compares the convergence speed and solution quality under different parameter combinations. The typical experimental results are shown in the following images and tables.

**Figure 8:**
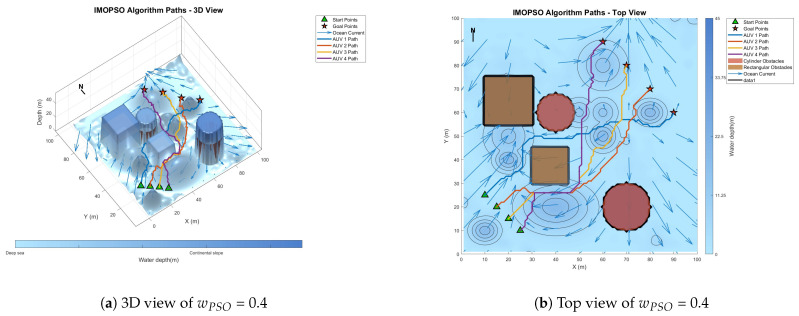
Global path planning of $w_{PSO}$ = 0.4.

**Figure 9:**
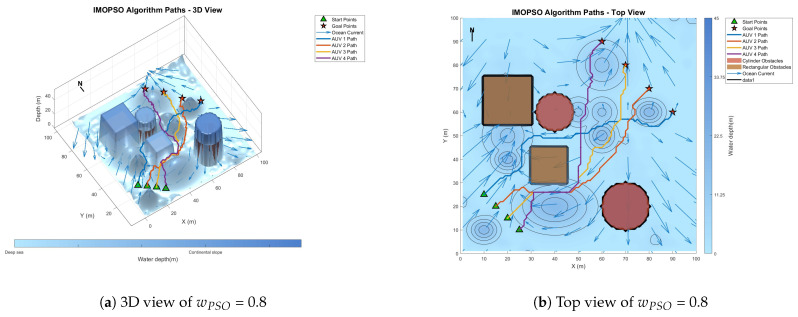
Global path planning of $w_{PSO}$ = 0.8.

**Figure 10:**
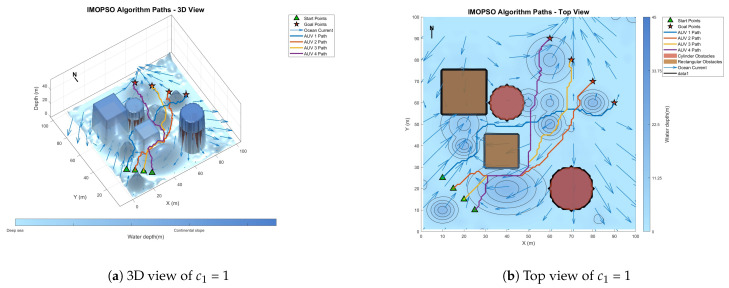
Global path planning of $c_{1}$ = 1.

**Figure 11:**
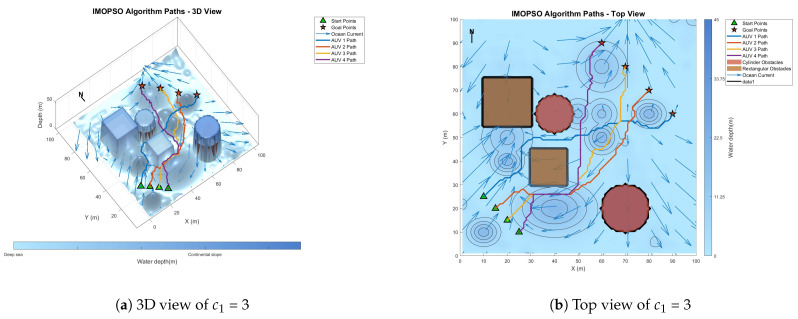
Global path planning of $c_{1}$ = 3.

**Figure 12:**
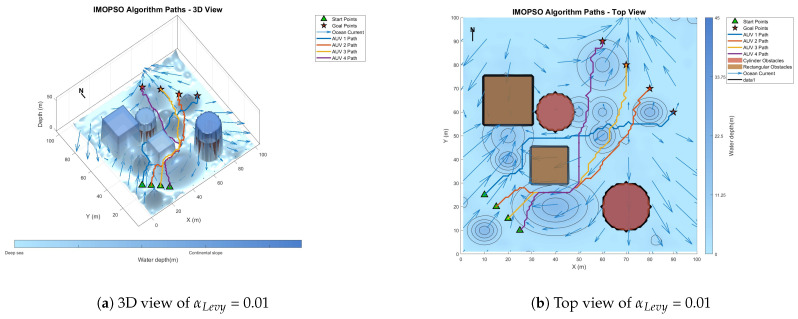
Global path planning of $\alpha_{Levy}$ = 0.01.

**Figure 13:**
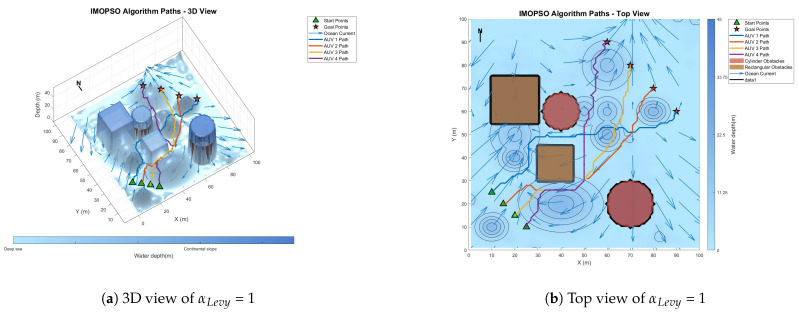
Global path planning of $\alpha_{Levy}$ = 1.

**Figure 14:**
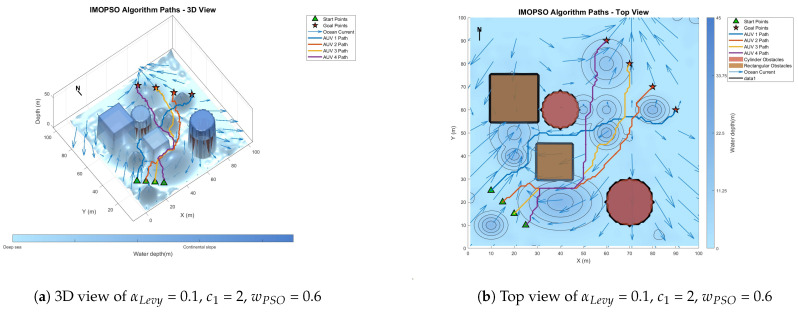
Global path planning of $\alpha_{Levy}$ = 0.1, $c_{1}$ = 2, $w_{PSO}$ = 0.6.

Figure 8 shows the global path-planning image when $w_{pso}$ = 0.4. Figure 9 shows the global path-planning image when $w_{pso}$ = 0.8. Figure 10 shows the global path-planning image when $c_{1}$ = 1. Figure 11 shows the global path-planning image when $c_{1}$ = 3. Figure 12 shows the global path-planning image when $\alpha_{Levy}$ = 0.01. Figure 13 shows the global path-planning image when $\alpha_{Levy}$ = 1. Figure 14 shows the global path-planning image when $w_{PSO}$ = 0.6, $c_{1}$ = 2, $\alpha_{Levy}$ = 0.1.

From the combined analysis of the images and Table 2, it can be seen that when $c_{1}$ = 1.0, the particles rely more on individual cognition, the path exploration is sufficient, but the convergence is slow. When $c_{1}$ = 3.0, the algorithm prematurely converges to a suboptimal solution, and the path length increases. When $c_{1}$ = 2.0, it reaches the optimal value, achieving a balance between exploration and exploitation and enabling the algorithm to converge rapidly and stably. When $\alpha_{Levy}$ = 0.01, the disturbance is insufficient, and path deadlock is prone to occur in complex terrain. When $\alpha_{Levy}$ = 1, the particle trajectory oscillates and the energy consumption increases. When $\alpha_{Levy}$ = 0.1, it reaches the optimal value. By applying moderate perturbations, the obstacle avoidance capability is enhanced and the safety margin is increased. When $w_{pso}$ = 0.8, unnecessary turns occur in the path; when $w_{pso}$ decreases from 0.6 to 0.4 using the dynamic attenuation strategy, it balances early exploration and late convergence. Therefore, in the experiment, the combination of $c_{1}$ = 2.0, $\alpha_{Levy}$ = 0.1 and $w_{pso}$ = 0.4 was adopted.

### 6.3. Comparative Experiment on Path Planning of IMOPSO Algorithm

**Figure 15:**
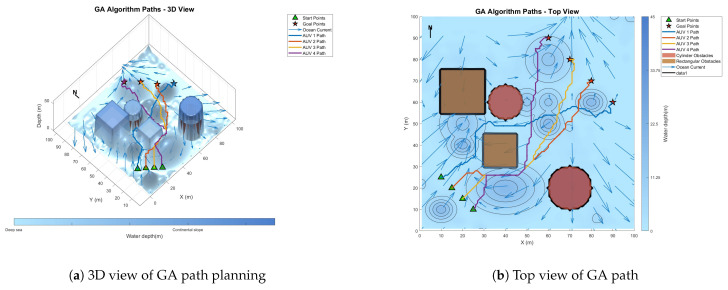
Global path planning GA.

**Figure 16:**
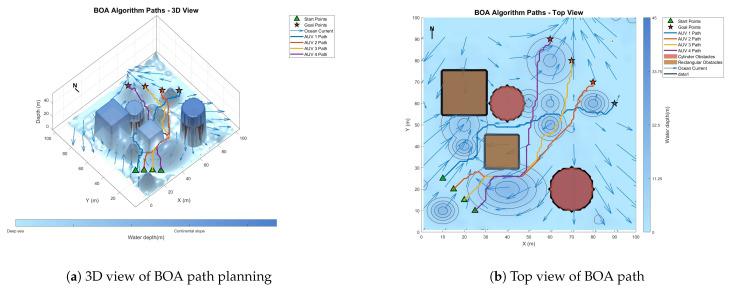
Global path planning of the BOA algorithm.

**Figure 17:**
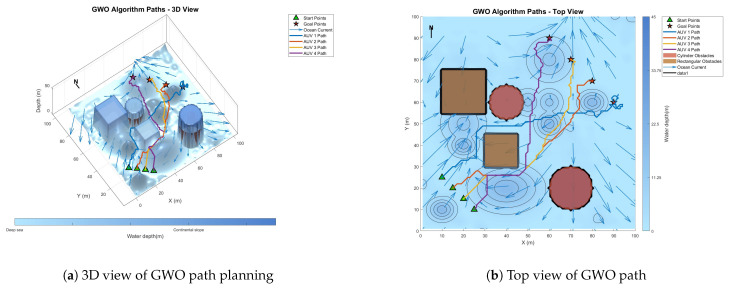
Global path planning GWO.

**Figure 18:**
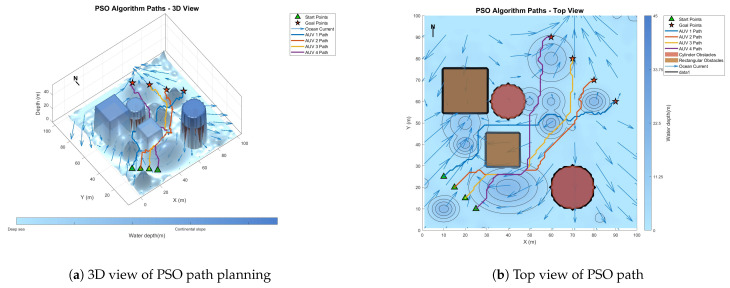
Global path planning PSO.

**Figure 19:**
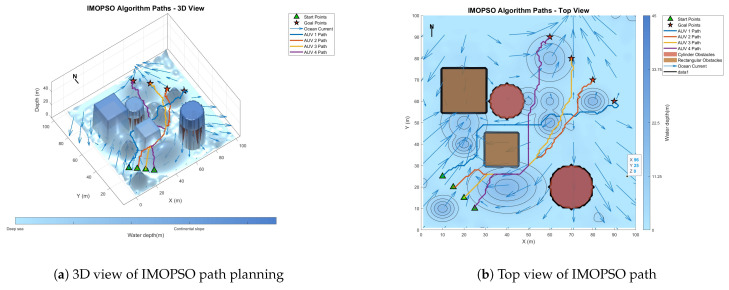
Global path-planning IMOPSO.

**Figure 20:**
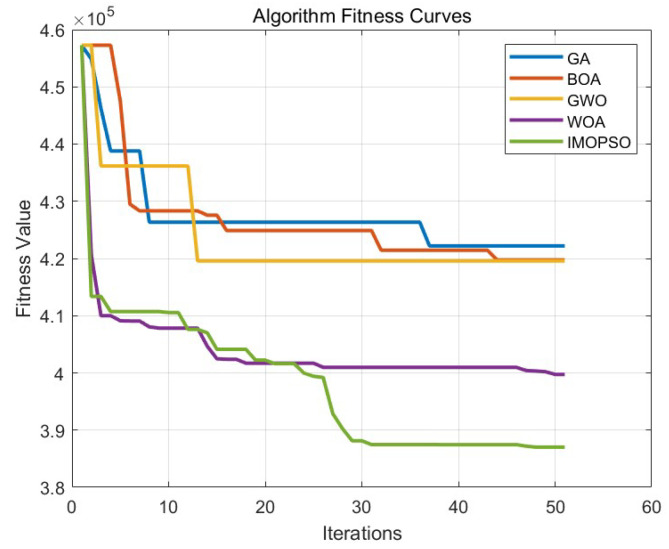
Global path-planning of IMOPSO algorithm and performance comparison.

To prove the effectiveness and accuracy of the improved multi-objective particle swarm optimization algorithm in global path planning under known environmental conditions, this paper takes the path-planning tasks of multiple AUVs in a three-dimensional marine environment as the research object and designs and implements a series of simulation experiments. The experimental content mainly includes the comparison of the GA algorithm, BOA algorithm, GWO algorithm, WOA algorithm, and IMOPSO algorithm. The comparison is mainly made from several aspects, such as path length, path energy consumption, path smoothness, and total fitness. Among them, Figure 15 is the top view and three-dimensional view of the global path-planning route of the GA algorithm. Figure 16 shows the top view and three-dimensional view of the global path-planning route of the BOA algorithm. Figure 17 shows the top view and 3D view of the global path-planning route of the GWO algorithm. Figure 18 shows the top view and 3D view of the global path-planning route of the PSO algorithm. Figure 19 shows the top view and 3D view of the global path-planning route of the IMOPSO algorithm. Figure 20 shows the fitness curves of the three algorithms. Table 3 presents the final results of several algorithms. As can be seen from the table, the improved multi-objective particle swarm optimization algorithm outperforms other meta-heuristic optimization algorithms in terms of path length, energy consumption, and path smoothness.

The results show that the path length and security performance of the IMOPSO algorithm are relatively outstanding. Compared with other meta-heuristic optimization algorithms, it can obtain the optimal objective function value and has a better iterative curve. The experimental results show that the IMOPSO algorithm proposed in this paper has a strong global search ability and a strong ability to avoid local optima. It can effectively search for the optimal path and obtain a better fitness function value.

### 6.4. Multi-AUV Dynamic Cooperative Path-Planning Experiment

**Figure 21:**
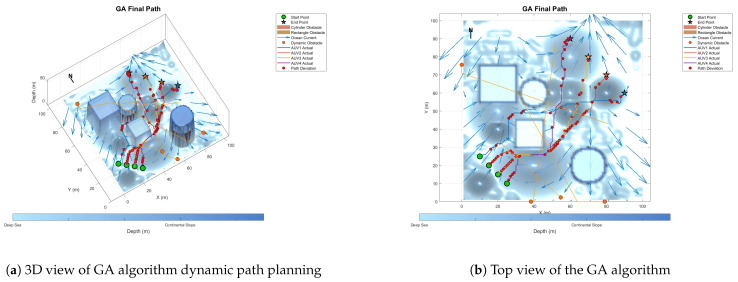
Dynamic path planning based on GA algorithm.

**Figure 22:**
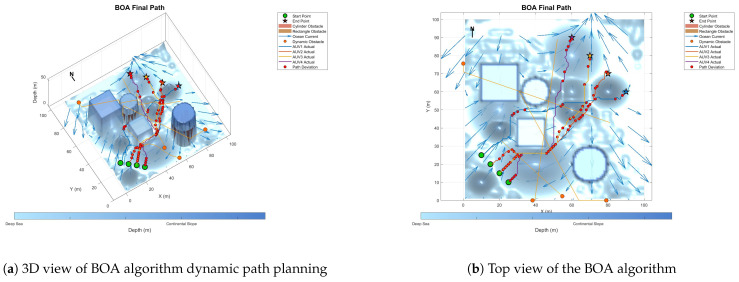
Dynamic path planning based on the BOA algorithm.

**Figure 23:**
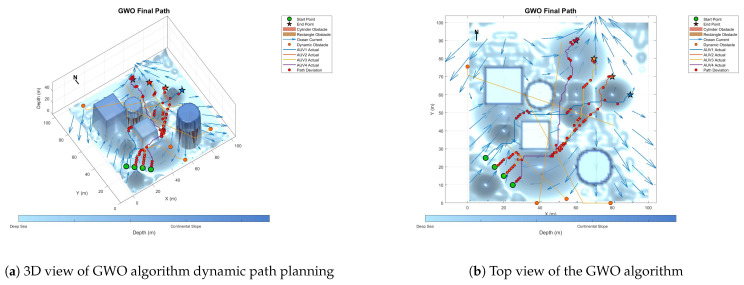
Dynamic path planning based on GWO algorithm.

**Figure 24:**
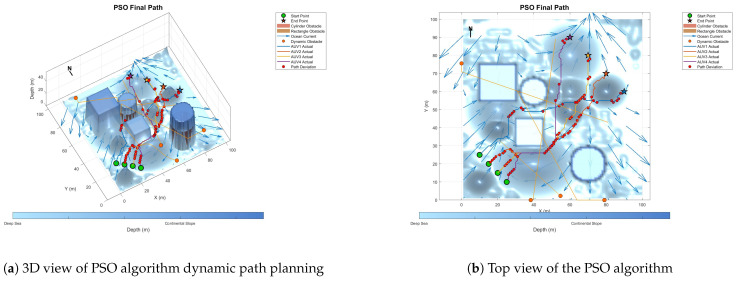
Dynamic path planning based on the PSO algorithm.

**Figure 25:**
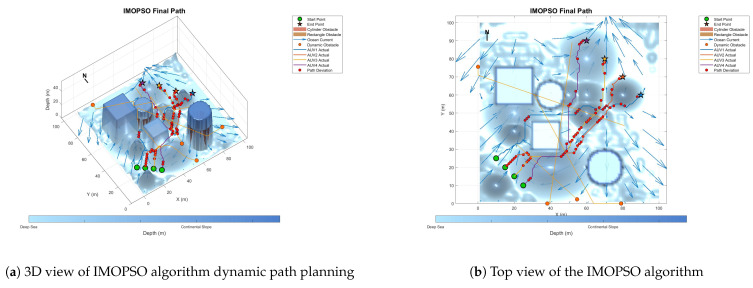
Dynamic path planning based on IMOPSO algorithm.

Because dynamic path planning involves multiple complex environmental factors, it is difficult to uniformly select evaluation indicators (such as path length, energy consumption, obstacle avoidance safety, etc.). Therefore, in this paper, the variation curve of the cost function with the number of iterations in the global path-planning optimization process is adopted as the comparison method to reflect the advantages and disadvantages of different algorithms in terms of convergence speed and stability. The dynamic path planning of the five algorithms is shown in the figure. Figure 21 Dynamic planning results of global path planning using the GA algorithm combined with the DWA algorithm. Figure 22 presents the dynamic programming results of global path planning using the BOA algorithm combined with the DWA algorithm. Figure 23 presents the dynamic programming results of global path planning using the GWO algorithm combined with the DWA algorithm. Figure 24 presents the dynamic programming results of global path planning using the WOA algorithm combined with the DWA algorithm. Figure 25 presents the dynamic programming results of global path planning using the IMOPSO algorithm combined with the DWA algorithm.

The red balls in the figure indicate the differences between the dynamic programming path and the static global programming path. It can be seen from the figure that the performance of the IMOPSO and DWA hybrid algorithm in dynamic path planning is superior to that of other meta-heuristic algorithms. Its advantages are mainly reflected in the early stage of iteration. The cost function of IMOPSO–DWA drops rapidly, indicating that it has a stronger global search ability and a higher initial convergence efficiency. Unlike the oscillation or even regression of the other meta-heuristic optimization algorithms’ curves in the middle and later stages, the cost curve of IMOPSO–DWA tends to stabilize after approaching the optimal solution, showing good robustness and stability. Under the same number of iterations, the final generated value obtained by the IMOPSO–DWA algorithm is significantly lower than that of several other algorithms, indicating that it can search for path solutions of better quality.

To sum up, it can be known that through the comparative analysis of the iterative curves of the cost function, the IMOPSO–DWA hybrid algorithm shows better optimization performance in the dynamic path-planning task, providing an effective solution idea for multi-AUV cooperative path planning in complex dynamic environments.

## 7. Conclusions

In today’s world, with the continuous growth of demands for marine resource development, environmental monitoring, and marine security, the autonomous path-planning ability of underwater AUVs in complex marine environments is becoming increasingly important. How to achieve efficient and safe path planning in a dynamic environment has become one of the key challenges of intelligent ocean systems.

This paper constructs a three-dimensional complex marine environment simulation platform integrating submarine topography, static obstacles, dynamic threats, and ocean current modeling, and proposes an improved multi-objective particle swarm optimization algorithm based on Chebyshev chaotic initialization for global path planning. Meanwhile, the improved Dynamic Window Method is introduced as the local obstacle avoidance compensation mechanism to achieve the fusion optimization of the global and local paths. The experimental results show that the proposed algorithm is superior to the traditional methods in terms of path feasibility, smoothness, convergence speed, and obstacle avoidance ability. Especially in multi-obstacle and strong ocean current environments, it shows stronger robustness and adaptability.

This study presents an effective multi-level solution for cooperative path planning of AUVs in complex and dynamic marine environments. Future work will focus on several key directions, including incorporating models of underwater communication delays and bandwidth constraints to optimize the real-time performance of multi-AUV collaborative decision-making; integrating reinforcement learning to enable dynamic task allocation and improve adaptability in complex scenarios; and embedding a real-time ocean turbulence prediction module to enhance the robustness of path planning against sudden flow field disturbances.

## Figures and Tables

**Table 1:** Parameter definitions and values.

Parameter Name	Parameter Definition	Value
*T*	Maximum Iterations	100
num_particles	Population size	100
knot_num	Path Nodes	10
*N*	Cubic spline interpolation points	200
$w_{max}$	Maximum inertia weight	0.9
$w_{min}$	Minimum inertia weight	0.4
$c_{1\alpha }$	$c_{1}$ lower boundary control factor	1
$c_{1\beta }$	$c_{1}$ upper boundary control factor	1.5
$c_{2\alpha }$	$c_{2}$ lower boundary control factor	1
$c_{2\beta }$	$c_{2}$ upper boundary control factor	1.5
$F_{max}$	Maximum difference weight	0.5
$F_{min}$	Minimum difference weight	0.1
$\beta_{0}$	Initial perturbation amplitude for local search	1
α	Exponential decay coefficient	4
*k*	Control parameter of sine chaotic perturbation	1200
$\delta_{0}$	Initial perturbation amplitude	10
$\Delta \omega_{base}$	Base angular velocity resolution	0.1
*K*	Velocity samples per step	50
$t_{resp}$	System response time	0.5
$\delta_{min}$	Minimum static safety margin	3
$d_{safe}$	Multi-AUV safety distance	5
$d_{com}$	Communication distance	50
η	Velocity sensitivity factor	0.8
$J_{max}$	Maximum jerk limit	2.0
$T_{p}$	Prediction horizon steps	10
α	Prediction-correction factor	0.7

**Table 2:** Results of parameter sensitivity experiments.

Parameters	Value	Iteration Count	Path Length	Energy
$c_{1}$	1	38	562.4	215.7
$c_{1}$	2	22	541.1	197.9
$c_{1}$	3	18	548.3	206.5
$\alpha_{Levy}$	0.01	18	558.2	208.3
$\alpha_{Levy}$	0.1	24	542.7	199.1
$\alpha_{Levy}$	1	29	551.6	211.8
$w_{PSO}$	0.4	32	552.9	205.4
$w_{PSO}$	0.6	23	543.3	200.2
$w_{PSO}$	0.8	27	547.5	209.7

**Table 3:** Comparison of path-planning performance of different algorithms.

Algorithm	Path Length	Energy	Smoothness	Threat Penalty	Total Fitness
GA	560.08	213.57	1.811	4214.24	422,197.93
BOA	536.04	184.48	1.569	4190.34	419,754.35
GWO	589.83	220.64	1.865	4187.64	419,574.23
PSO	549.54	190.54	1.472	3991.32	399,736.68
IMOPSO	463.84	200.28	1.562	3862.72	387,020.33

## Data Availability

The data presented in this study are available in the main text.

## Abbreviations

The following abbreviations are used in this manuscript:

AUV	Autonomous Underwater Vehicle
PSO	Particle Swarm Optimization
DWA	Dynamic Window Approach
GA	Genetic Algorithm
